# Chyluria secondary to disseminated tuberculosis in a 13‐year‐old female child: A case report

**DOI:** 10.1002/ccr3.8169

**Published:** 2023-11-13

**Authors:** Mukesh Bhatta, Rejeena Subedi, Abhishek Shah, Ranjita Baral, Lalan Prasad Rauniyar, Shishir Shrestha, Asha Ghimire

**Affiliations:** ^1^ Department of Pediatrics and Adolescent Medicine B.P. Koirala Institute of Health Sciences Dharan Nepal

**Keywords:** chyle, filariasis, milky urine, tuberculosis

## Abstract

We report the case of chyluria secondary to disseminated tuberculosis in a 13‐year‐old female child who presented with passage of white colored urine since 5 months, progressive weight loss for 3 months, abdominal distension for 2 months, generalized swelling of body for 15 days, and pain in abdomen for 10 days. Child had good recovery following treatment with antitubercular drugs. Though chyluria is uncommon in children, tuberculosis could be considered as a differential, after ruling out filariasis.

## 1 INTRODUCTION

The passage of milky white substance “chyle” in urine is called chyluria. It occurs as a result of an abnormal communication between the lymphatic and urinary systems. Chyluria remains endemic in parts of Asia, especially India, and also Sub‐Saharan Africa.^1^ Chyluria can be parasitic or nonparasitic in origin. Majority of parasitic causes are due to lymphatic filariasis, with the nematode *Wuchereria bancrofti* contributing to more than 90% of all cases. Non parasitic causes are rarer and these are almost always non tropical.^2^, ^3^ Other parasitic causes of chyluria include cysticercosis, ascariasis, and echinococcosis. Aetiological factors include thoracic duct abnormalities, retroperitoneal abscess, tuberculosis, malignancy, pregnancy, aortic aneurysm, blunt, or penetrating trauma, as a complication of surgery such as partial nephrectomy or aorto‐iliac bypass, lymphatic malformation, radiation, and congenital abnormalities.^4^, ^5^, ^6^ Chyluria in children is very rare and under‐reported, with the majority being described in case reports.^7^ The main constituent of chyle is dietary lipids, proteins, and fat‐soluble vitamins.^8^ Chyluria may be associated with dysuria or hematuria due to rupture of small blood vessels adjacent to the fistulous communication.^9^, ^10^ Patients may have anemia, hypoproteinemia due to loss of protein, and fat in the urine subsequent to weight loss and malnutrition.^8^, ^11^, ^12^ Chyluria can be confused with nephrotic syndrome when massive proteinuria is present on urine examination during evaluation of milky or white urine.^8^

## 2 CASE HISTORY AND EXAMINATION

Here we present a case of a 13‐year‐old female patient who developed chyluria secondary to disseminated tuberculosis, and subsequently responded well to antitubercular drugs.

A 13‐year‐old, well immunized, female patient was brought to the pediatric emergency of B.P. Koirala Institute of Health Sciences, Dharan, Nepal with complaints of passage of white colored urine since 5 months, progressive weight loss for 3 months, abdominal distension for 2 months, generalized swelling of body for 15 days, and pain in abdomen for 10 days.

**FIGURE 1:**
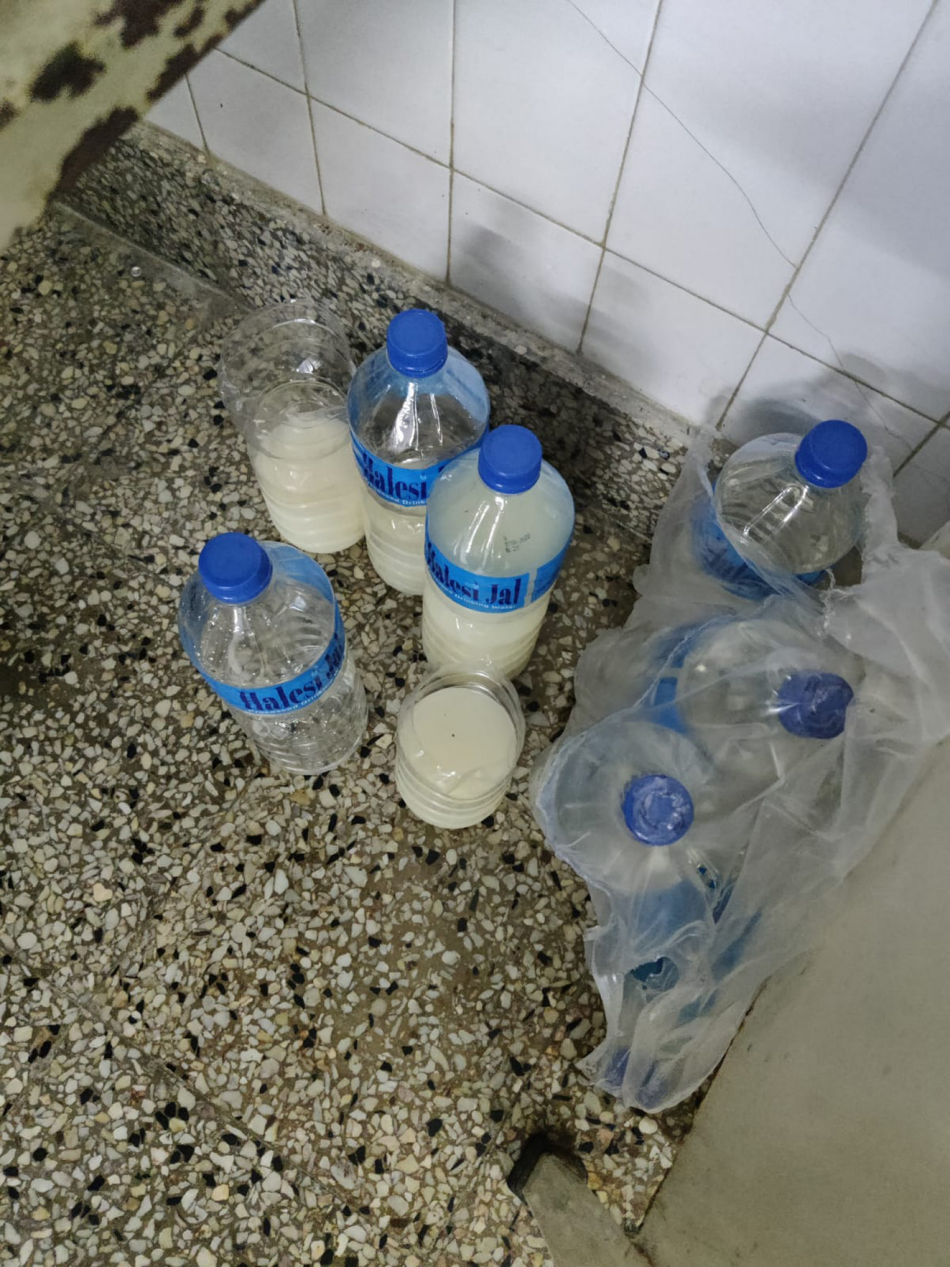
Milky white color of urine suggesting chyluria.

The color of the urine was milky white, as shown in Figure 1. There was no history of pain during micturition or increased frequency of micturition. There was no history of fever. The child had lost appetite and had lost her weight significantly since the onset of illness. The abdominal distension was generalized, not associated with pain initially. For the last 15 days, the child started having generalized swelling of the body starting from the face and then progressing to the limbs. There was history of frothy urine. However the urine output did not decrease. After the onset of anasarca, there was abdominal pain, generalized, and moderate to severe in intensity. The abdominal pain was associated with vomiting. The bowel movement was normal. The patient initially took some oral analgesics and oral antibiotics from local practitioners; however, the condition did not improve. There is history of pulmonary tuberculosis in her grandmother 3 years back, for which she completed a course of 6 months of antitubercular drugs, and currently she is doing well. There is no history of similar illness in the family, or any other sick contacts recently. There is no history of travel within the last 6 months. There is no history of fever, cough, and difficulty breathing. There is no history of prolonged drug intake, substance abuse, joint pain, joint swelling, rashes, or any neuropsychiatric symptoms. There is no history of blood transfusion or any sort of surgical interventions in the past. The birth history and developmental history is normal. She belongs to a low socioeconomic status.

On examination at presentation, the vitals were stable. There was no pallor, icterus, clubbing, cyanosis, or dehydration. There was lymphadenopathy present, notably in the anterior, and posterior cervical region. The lymph nodes were matted, non‐tender and largest measured 2 cm × 1 cm. The weight of the child was 31 kg and height 155 cm, with a body mass index (BMI) of 13.3 kg/m^2^. On examining the abdomen, there was moderate ascites with generalized tenderness all over the abdomen, suggestive of ascites with spontaneous bacterial peritonitis. There was no organomegaly. The genitalia were normal. The respiratory system examination revealed decreased breath sounds over bilateral lung bases. Cardiac and neurological systems were well within normal limits.

### 2.1 Differential diagnosis, investigations and treatment

The child was admitted with a working diagnosis of chyluria with moderate ascites with spontaneous bacterial peritonitis. Lymphatic filariasis, abdominal tuberculosis and malignancy were considered as differentials, and investigations sent. The patient had mild anemia. The renal function, lipid profile, complement levels, serum ANA, dsDNA, serum phosphorous, amylase, and lipase levels were normal. The peripheral smear for microfilaria was negative. The microfilariae antigen test (immunochromatographic test) was negative. Similarly, dengue serology, scrub typhus serology, and malarial serology were negative. Lymphangiography could not be done due to nonavailability. The details of the investigation reports are presented in Tables 1, 2, 3.

**TABLE 1:** Hematological and biochemical laboratory investigations.

SN	Tests	Reports
1.	Hemoglobin	9.8 g/dL
2.	Total leukocyte count	11,520 cells/mm3
3.	Differential leukocyte count	N84%, L 10%, M 05%, E 1%
4.	AEC count	250 cells/ml
5.	Platelets	1,66,000 cells/mm3
6.	Urea/creatinine	11.2/0.5 mg/dL
7.	Sodium/potassium	131/2.8, after repeating 137/4.3 meq/L
8.	Liver function test	Total protein: 3.23 g/dL
		Albumin: 1.71 g/dL
		Total bilirubin: 0.4 mg/dL
		ALT: 11/AST: 21/ALP:55 U/L
9.	Lipid profile (mg/dl)	Total cholesterol: 112.59; HDL‐cholesterol: 34.16; LDL cholesterol: 73.94
10.	Urine	Chyle positive; triglyceride:121.35 mg/dL
11.	Peripheral smear for microfilaria	Negative
12.	Microfilariae antigen test‐immunochromatographic test	Negative
13.	Peripheral smear for general blood picture	Normocytic to microcytic hypochromic cells
14.	Ascitic fluid	Glucose: 108 mg/dL, protein 0.5 g/dL, TLC: plenty, granulocyte: 60, non‐granulocyte 20, RBC: plenty; ADA: 20 IU/L
15.	Ascitic fluid culture	Sterile
16.	Urine RE/ME	Protein: 3+
		Sugar: 1+
		WBC: 2–4
		RBC: plenty
17.	24 h urine protein	>40 mg/m^2^/h
18.	Spot urine protein: creatinine ratio	2.27
19.	Urine culture and sensitivity	Acinetobacter baumannii complex; sensitive to piperacillin tazobactam; resistant to: amikacin, cefepime, cefotaxime, ceftriaxone, nitrofurantoin
20.	Blood culture	Sterile after 48 h of aerobic incubation
21.	HIV/HBSAg/HCV	Negative

**TABLE 2:** Tuberculosis work up.

SN	Test	Report
1.	Mantoux test	Positive (13 mm after 48 h)
2.	Sputum for AFB staining	Negative
3.	Gene expert (sputum, ascitic fluid and pleural fluid)	MTB not detected
4.	FNAC of mesenteric lymph nodes	Smear examined shows predominant lymphocytes scattered against lipoproteinaceous background. In view of whitish nature of fluid aspirated and cytological findings, chyle is considered.

**TABLE 3:** Radiological work up.

SN	Test	Report
1.	Ultra sound abdomen with KUB	Bilateral mildly echogenic kidneys. Right nephrolithiasis 3.7 mm noted in upper pole of calyx Few prominent large bowel loops with surrounding omentum‐? Inflammatory colitis Splenic cyst
2.	CECT abdomen and pelvis	Multiple enhancing and some non‐enhancing necrotic para aortic, peripancreatic and mesenteric lymph nodes? tubercular pathology Omental and mesenteric fat stranding in lower abdomen Diffuse long segment of thickened large bowel wall involving descending colon, sigmoid and rectum with mucosal enhancement and submucosal edema and low attenuation of muscularis layer indicative of? acute colitis Hypoattenuating and hypoenhancing and bulky head and uncinate process of pancreas with peripancreatic fat stranding Minimal ascites Two well‐defined nonenhancing cystic lesions with no internal septal calcification in splenic parenchyma‐likely splenic cyst.

Considering a positive family history of tuberculosis along with features like ascites, weight loss, and anasarca, along with investigation parameters like positive mantoux test, presence of multiple enhancing and some non‐enhancing necrotic para aortic, peripancreatic and mesenteric lymph nodes with omental and mesenteric fat stranding in lower abdomen in CECT abdomen and a negative microfilaria test, a diagnosis of “chyluria secondary to disseminated tuberculosis with culture positive urinary tract infection with spontaneous bacterial peritonitis with nephrotic range proteinuria with anemia (probably nutritional) with hypokalemia with undernutrition,” was made.

### 2.2 Treatment received and patient course

The patient was admitted to the pediatric ward and intravenous fluids and electrolytes started. Ultra sound guided tapping of ascitic and pleural fluid was done and sent for analysis. Initially injection ceftriaxone at the rate of 75 mg/kg/day was started, in view of spontaneous bacterial peritonitis. Later, antibiotic was changed to injection piperacillin‐tazobactam at the rate of 100 mg/kg/dose three times a day after seeing urine culture sensitivity reports. Antibiotics were given for 7 days. Tuberculosis work up was done, as shown in Table 2. Ultra sound guided Fine Needle Aspiration Cytology (FNAC) of mesenteric lymph nodes was done. In view of ongoing chyluria, diet rich in medium chain triglycerides and high protein was provided after consultation with dietitian. Multivitamin supplements with fat‐soluble vitamins were given. The child was started on antitubercular treatment as per the national tuberculosis guideline consisting of 2 months of isoniazide, rifampicin, pyrazinamide, and ethambutol, followed by 10 months of isoniazide and rifampicin. The child was also started on oral iron therapy.

### 2.3 Outcome and follow up

The abdominal pain subsided after 2 days of starting of intravenous antibiotics. Patient started to take orally and was discharged on antitubercular drugs. Chyluria was present during discharge. On follow‐up after 2 months of starting of antitubercular treatment, chyluria stopped, swelling decreased, appetite regained, and patient experienced a well‐being along with weight gain of two kilograms. There was no proteinuria and edema at this follow up. The child is in regular follow up, and the next follow‐up is scheduled after 1 month.

## 3 DISCUSSION

Lymphatic filariasis is the most common cause of chyluria. Since it takes years to manifest clinically, lymphatic filariasis is rare in children. However, there are other etiologies apart from filariasis which may cause chyluria. Conditions like thoracic duct abnormalities, retroperitoneal abscess, tuberculosis, malignancy, and congenital abnormalities may rarely cause chyluria. Sometimes it may manifest like nephrotic syndrome with massive proteinuria; however the serum cholesterol, and albumin levels are normal in chyluria, unlike nephrotic syndrome.^4^, ^5^, ^6^ Therefore it is crucial to differentiate chyluria from nephrotic syndrome to prevent unnecessary immunosuppression treatment.

There have been very few cases reported of chyluria in children secondary to tuberculosis. Dev Nishanth and Kumar Rahul reported chyluria secondary to tuberculosis in a 30‐year‐old female patient.^13^ However, case reports of chyluria in children secondary to tuberculosis are very rare. In another report by Wilson, R. S. E. and White, R. J, a 25–year‐old female has been reported to have chyluria secondary to lymph node tuberculosis.^14^ As per a case reported by Le Dantrec, chyluria was associated with tubercular peritonitis. This was cited in the article by Wilson, R. S. E. and White, R. J.^14^ The chyle is thought to reach the urine via a lymphaticourinary fistula. When the lymphatics get blocked, there is formation of lymphatic varices, which ultimately rupture and the lymph thus passes to the renal calyces, ureters, bladder, or even urethra.^15^ The lymph is rich in albumin, and this passage of lymph in urine manifests as nephrotic range proteinuria, as seen in our case, mimicking nephrotic syndrome.

This case had a positive family history of contact with pulmonary tuberculosis, along with a positive mantoux test and typical CECT findings of abdominal tuberculosis. These features combined with history of weight loss and anorexia suggests a tubercular etiology. Further, the patient responded well to antitubercular therapy. The possible mechanism behind chyluria could be formation of fistulous tracts secondary to extensive tubercular inflammation and necrosis. Early diagnosis and treatment is crucial as this may prevent possible complications like dehydration and malnutrition.

## 4 CONCLUSION

Chyluria in children is very rare. Though lymphatic filariasis is the most common cause of chyluria, other common etiologies like tuberculosis, malignancy, and congenital duct abnormalities should be ruled out. Tuberculosis, as an etiology could be considered in children, especially in this part of the world, presenting as chyluria. Early institution of antitubercular therapy is crucial in preventing complications.

## AUTHOR CONTRIBUTIONS

**Mukesh Bhatta:** Conceptualization; data curation; formal analysis; investigation; methodology; project administration; resources; software; supervision; validation; visualization; writing – original draft; writing – review and editing. **Rejeena Subedi:** Conceptualization; data curation; formal analysis; methodology; software; validation; writing – original draft; writing – review and editing. **Abhishek Shah:** Formal analysis; investigation; methodology; resources; software; visualization; writing – original draft. **Ranjita Baral:** Data curation; formal analysis; investigation; methodology; resources; software; writing – review and editing. **Lalan Prasad Rauniyar:** Data curation; formal analysis; investigation; methodology; resources; software; supervision; writing – review and editing. **Shishir Shrestha:** Data curation; formal analysis; investigation; methodology; resources; software; validation. **Aasha Ghimire:** Data curation; formal analysis; investigation; methodology; software; writing – review and editing.

## FUNDING INFORMATION

No funding was obtained for this study.

## CONFLICT OF INTEREST STATEMENT

The authors declare that they have no competing interests.

## CONSENT

Written informed consent was obtained from the patient's mother to publish this report in accordance with the journal's patient consent policy.

## REGISTRATION OF RESEARCH STUDIES

This is a case report so registration was not required.

## GUARANTOR

All the authors are the guarantor of the study.

## PROVENANCE AND PEER REVIEW

Not commissioned or externally peer‐reviewed.

## Data Availability

The datasets supporting the conclusions of this article are included within the article.
